# Creatine homeostasis and protein energy wasting in hemodialysis patients

**DOI:** 10.1186/s12967-021-02780-y

**Published:** 2021-03-20

**Authors:** Adrian Post, Joëlle C. Schutten, Daan Kremer, Yvonne van der Veen, Dion Groothof, Camilo G. Sotomayor, Christa A. Koops, Pim de Blaauw, Ido P. Kema, Ralf Westerhuis, Theo Wallimann, M. Rebecca Heiner-Fokkema, Stephan J. L. Bakker, Casper F. M. Franssen

**Affiliations:** 1 Department of Internal Medicine, University of Groningen, University Medical Center Groningen, 9713 GZ Groningen, The Netherlands; 2 Department of Laboratory Medicine, University of Groningen, University Medical Center Groningen, Groningen, 9713 GZ the Netherlands; 3 Dialysis Center Groningen, 9713 GZ Groningen, The Netherlands; 4 Department of Biology, ETH Zurich, Zurich, Switzerland

**Keywords:** Creatine, Guanidinoacetate, Arginine, Protein energy wasting, Muscle mass, Fatigue, Hemodialysis

## Abstract

Muscle wasting, low protein intake, hypoalbuminemia, low body mass, and chronic fatigue are prevalent in hemodialysis patients. Impaired creatine status may be an often overlooked, potential contributor to these symptoms. However, little is known about creatine homeostasis in hemodialysis patients. We aimed to elucidate creatine homeostasis in hemodialysis patients by assessing intradialytic plasma changes as well as intra- and interdialytic losses of arginine, guanidinoacetate, creatine and creatinine. Additionally, we investigated associations of plasma creatine concentrations with low muscle mass, low protein intake, hypoalbuminemia, low body mass index, and chronic fatigue. Arginine, guanidinoacetate, creatine and creatinine were measured in plasma, dialysate, and urinary samples of 59 hemodialysis patients. Mean age was 65 ± 15 years and 63% were male. During hemodialysis, plasma concentrations of arginine (77 ± 22 to 60 ± 19 μmol/L), guanidinoacetate (1.8 ± 0.6 to 1.0 ± 0.3 μmol/L), creatine (26 [16–41] to 21 [15–30] μmol/L) and creatinine (689 ± 207 to 257 ± 92 μmol/L) decreased (all P < 0.001). During a hemodialysis session, patients lost 1939 ± 871 μmol arginine, 37 ± 20 μmol guanidinoacetate, 719 [399–1070] μmol creatine and 15.5 ± 8.4 mmol creatinine. In sex-adjusted models, lower plasma creatine was associated with a higher odds of low muscle mass (OR per halving: 2.00 [1.05–4.14]; P = 0.04), low protein intake (OR: 2.13 [1.17–4.27]; P = 0.02), hypoalbuminemia (OR: 3.13 [1.46–8.02]; P = 0.008) and severe fatigue (OR: 3.20 [1.52–8.05]; P = 0.006). After adjustment for potential confounders, these associations remained materially unchanged. Creatine is iatrogenically removed during hemodialysis and lower plasma creatine concentrations were associated with higher odds of low muscle mass, low protein intake, hypoalbuminemia, and severe fatigue, indicating a potential role for creatine supplementation.

**Supplementary Information:**

The online version contains supplementary material available at 10.1186/s12967-021-02780-y.

## Clinical perspectives

- Creatine homeostasis in hemodialysis patients is largely unknown, but impaired creatine homeostasis may contribute to protein energy wasting.
- Arginine, guanidinoacetate, creatine and creatinine all demonstrated an intradialytic decrease in plasma concentrations. We quantified the intradialytic losses and demonstrated that the majority of creatine is removed from the intracellular compartment during hemodialysis.
- We demonstrated that lower plasma creatine concentrations are associated with higher odds of low muscle mass, low protein intake, hypoalbuminemia, and severe fatigue.

## Introduction

Managing the nutritional aspects of chronic kidney disease (CKD) presents a wide variety of challenges. While overnutrition is a major problem in the general population, patients with CKD, especially those depending on hemodialysis treatment, are more prone to protein energy wasting (PEW), a state of decreased body stores of protein and energy fuels [1, 2]. Muscle wasting, low protein intake, hypoalbuminemia, low body mass and chronic fatigue are very prevalent in hemodialysis patients, with major impacts on the quality of life and longevity [1–3].

An impaired creatine status may be a generally overlooked potential contributor to symptoms related to PEW in hemodialysis patients. Creatine is a natural nitrogenous organic acid that is integral to energy metabolism and crucial for proper cell functioning [4]. In humans, roughly 90% of all creatine and its high-energy product phosphocreatine are located inside skeletal muscles where it serves as an energy buffer par excellence [5]. Yet, on a daily basis, roughly 1.6 to 1.7% of the total creatine pool is degraded to creatinine, necessitating a continuous replenishment of the pool with new creatine [6]. Generally, in omnivores, approximately 50% of the daily requirement of creatine is endogenously synthesized and the rest is taken up through the diet from alimentary sources, like fresh meat and fish [7]. Importantly, however, the first, and rate-limiting step of the endogenous synthesis of creatine is facilitated primarily by the kidneys, where the enzyme arginine:glycine amidinotransferase (AGAT) converts arginine into guanidinoacetate [7–14]. Subsequently, guanidinoacetate is converted into creatine in the liver by the enzyme guanidinoacetate *N*-methyltransferase (GAMT) [9]. Given the fact that the rate limiting step of creatine synthesis depends on kidney function, and provided that hemodialysis patients virtually have no kidney function, it has been hypothesized that low endogenous creatine biosynthesis contributes to low creatine status and development of PEW in hemodialysis patients [7, 8]. Additionally, there may be unopposed losses of creatine and its precursors to the dialysate during every hemodialysis session. However, literature on creatine homeostasis in hemodialysis patients is scarce and the intradialytic losses have not been quantified.

The goals of the current study were (1) to explore creatine homeostasis during hemodialysis treatment and (2) to investigate whether plasma creatine concentrations are associated with characteristics of the PEW phenotype. To achieve the first goal, we assessed intradialytic plasma changes as well as intra- and interdialytic losses of creatine, its precursors arginine and guanidinoacetate, and its metabolite creatinine. To achieve the second goal, we investigated whether plasma creatine concentrations were associated low muscle mass, low protein intake, hypoalbuminemia, low body mass index, and chronic fatigue.

## Material and methods

### Design and study population

This observational study was performed according to ethical standards laid down in the 1964 Declaration of Helsinki and its later amendments and was approved by the Medical Ethical Committee of the University Medical Center Groningen, The Netherlands. All participating patients gave written informed consent. The methods of the study design have been described previously [15–17]. In short, inclusion criteria were twice or thrice weekly hemodialysis with 3–5 h per treatment, a hemodialysis vintage of ≥ 2 months, and absence of clinical signs of infection. Patients on a thrice weekly hemodialysis schedule dialyzed on either Monday–Wednesday–Friday or on Tuesday–Thursday–Saturday. In both cases the mid-week hemodialysis session was used in this study. For patients dialyzing twice weekly, the last hemodialysis session of the week was used.

In adherence to the Kidney Disease Outcomes Quality Initiative and national guidelines, our center applies a fistula first approach for vascular access creation, thereby starting with creation of a fistula as distally in the arm as possible [18]. The non-dominant arm was preferred to create the vascular access. In case of lower-arm arteriovenous fistulae, patients received a radiocephalic arteriovenous fistula. In case of upper-arm arteriovenous fistulae, patients received a brachiocephalic arteriovenous fistula or basilic vein transposition. In patients with previous maturation problems of fistulae, or patients with a subacute indication for dialysis (i.e. < 4 weeks), an arteriovenous graft was considered instead of the fistula first approach. The arteriovenous graft was a standard wall polytetrafluorethylene graft (Gore-Tex, WL Gore & Associates, Flagstaff, Arizona, USA), in either a loop or straight configuration. In patients without an arteriovenous fistula or an arteriovenous graft, a central venous catheter was used.

Hypertension was defined as predialysis systolic blood pressure ≥ 140 mmHg and/or diastolic blood pressure ≥ 90 mmHg. Medication usage and a history of cardiovascular disease and diabetes was obtained from the patients’ medical records. Cardiovascular disease was defined as a history of ischemic heart disease, congestive heart failure, coronary artery bypass grafting, percutaneous coronary intervention, stroke, or peripheral vascular disease. Blood pressure and weight were measured before and after hemodialysis. Body mass index (BMI) was defined as body weight postdialysis divided by the square of body height. Body surface area (BSA) was calculated using the formula of Du Bois and Du Bois [19]. All patients were asked to fill in the Checklist Individual Strength (CIS). The CIS is a self-reported multidimensional instrument to assess four qualitatively different aspects of fatigue (fatigue severity, concentration problems, reduced motivation, and reduced activity level). The CIS-questionnaire enquires about fatigue and fatigue-related behavioral aspects and consists of 20 statements for which the participant indicates on a 7-point Likert-scale to what extent the statement applies to the participant. CIS has been well-validated and is frequently used in research in patients with various illnesses [20–24], including dialysis patients [17, 25].

### Hemodialysis settings

All studies were performed with the Fresenius 5008 hemodialysis apparatus with a low-flux dialyzer (Fresenius Medical Care, Bad Homburg, Germany) using smart*b*ag dialysate concentrations (Fresenius Medical Care), as previously described [15–17]. Blood flow and dialysate flow were between 200 and 300 mL/min and between 500 and 700 mL/min, respectively. Dialysate temperature was 36.0 or 36.5 ºC. Dialysis fluid sodium varied from 136 to 140 mmol/L, potassium from 1 to 3 mmol/L, depending on the plasma potassium concentration, calcium varied from 1.25 to 1.50 mmol/L and bicarbonate from 34 to 38 mmol/L.

### Sample collection and laboratory measurements

During the hemodialysis session, all dialysate was collected in a 200-L tank. The total dialysate volume was measured by calculating the weight difference of the tank before and after the hemodialysis session. At the end of hemodialysis, all dialysate was homogenized, and samples were taken for analysis [15]. Blood was drawn directly from the dialysis line, at the start of hemodialysis and 5 min before the end of the hemodialysis session. Patients with significant residual diuresis, defined as a urine production of more than 200 ml/24-h, were asked to collect two 24-h urine collections before the hemodialysis session during which the dialysate was collected. For patients with a thrice-weekly hemodialysis schedule this was the complete interdialytic urine production.

Plasma and dialysate concentrations of arginine were measured using a LC–MS/MS method. In short, tandem mass spectrometry with hydrophilic interaction liquid chromatography (HILIC) was used to quantify the concentrations of arginine. Electrospray ionization (ESI) in positive mode has been used to monitor the transitions of arginine 175- > 116 and stable isotope labeled arginine (^13^C_6_-^15^N_4_-arginine) 185- > 122 as an internal standard.

Intra-assay and inter-assay coefficients of variation were 6.0% and 6.1% at 23 µmol/L and 3.9% and 5.3% at 492 µmol/L, respectively. Urinary arginine was measured using cation exchange chromatography with post column derivatisation (Biochrom Ltd.), separation was achieved by increasing the pH. Intra-assay and inter-assay coefficients of variation were 6.5% and 7.6% at 28 µmol/L. Plasma, dialysate, and urinary concentrations of guanidinoacetate and creatine were measured using a separate LC–MS/MS method. In short, tandem mass spectrometry reversed phase chromatography (RP-C18) was used to quantify the concentrations of creatine and guanidinoacetate. Electrospray ionization (ESI) in positive mode has been used to monitor the transitions of guanidinoacetate 118- > 76 and creatine 132- > 90, using stable isotope labeled guanidinoacetate (^13^C_2_-guanidinoacetate) 120- > 78 and stable isotope labeled creatine (D3-creatine) 135- > 93 as internal standards. For guanidinoacetate, intra-assay and inter-assay coefficients of variation were 2.9% and 6.1% at 2.8 µmol/L and 1.9% and 2.7% at 73 µmol/L, respectively. For creatine, intra-assay and inter-assay coefficients of variation were 3.9% and 6.5% at 17 µmol/L and 1.7% and 1.7% at 1065 µmol/L, respectively. Plasma creatinine, urea, albumin and high-sensitivity C-reactive protein were measured on Roche routine chemistry analyzers (Modular P/Cobas C, Roche Diagnostics, Mannheim, Germany). Interleukin-6 (IL-6) was measured in a subset of the participants (n = 39) using a Human IL-6 Quantikine HS Elisa kit (R&D systems, Minneapolis, United States). Other laboratory measurements were performed with automated and validated routine methods (Roche Diagnostics, Mannheim, Germany). The Kt/V was used to express hemodialysis efficacy and was calculated according to formula of Daugirdas [26]:

$$\text{Kt}/\text{V}= -\text{ln} (\text{R}-0.008 * t+(4-3.5 * \text{R}) * \text{UF}/\text{W}$$

in which R is the ratio between the post- and predialysis concentration of urea, t is duration of the hemodialysis session (h), UF is the ultrafiltration volume (L) and W the body weight after hemodialysis (kg).

### Intradialytic plasma changes

Intradialytic plasma changes of arginine, guanidinoacetate, creatine and creatinine are assessed by calculating the absolute decrease, as well as the proportional decrease:

Absolute decrease (μmol/L) = Predialysis concentration (μmol/L) – Postdialysis concentration (μmol/L). Proportional decrease (%) = Absolute decrease (μmol/L) / Predialysis concentration (μmol/L) * 100%.

### Losses of arginine, guanidinoacetate, creatine and creatinine during hemodialysis

To determine the losses of arginine, guanidinoacetate, creatine and creatinine to the dialysis fluid, the concentration of individual analytes was multiplied by the total volume of the spent dialysate: Total removal (in μmol) = V_Dialysate_ * D_X._ V_Dialysate_ refers to the total volume of the spent dialysate (L) and D_X_ refers to the measured concentration of arginine, guanidinoacetate, creatine and creatinine in the collected dialysate (μmol/L). The total losses per dialysis of arginine, guanidinoacetate, creatine and creatinine can be split into the losses from the extracellular space and the losses from the intracellular space after being shifted towards the extracellular space during the hemodialysis. The estimated extracellular losses of arginine, guanidinoacetate, creatine or creatinine were calculated using the pre- and postdialysis plasma concentrations of arginine, guanidinoacetate, creatine and creatinine and estimates of the pre- and postdialysis extracellular volume (ECV): Estimated extracellular losses (μmol) = P_X predialysis_ * ECV_predialysis_ – P_X postdialysis_ * ECV_postdialysis_. P_X predialysis_ and P_X postdialysis_ refer to predialysis and postdialysis plasma concentrations of arginine, guanidinoacetate, creatine and creatinine, respectively. In these calculations, the assumption is made that these small molecular solutes can freely diffuse throughout the extracellular fluid. ECV_predialysis_ and ECV_postdialysis_ were calculated using the formula by Abraham et al. (2011), defined as the square root of pre- or postdialysis body weight, multiplied by height [27]. Additionally, as a sensitivity analysis, the extracellular volume was also calculated using the formula by Bird et al. [28]: ECV = weight^0.6469^ × height^0.7236^ × 0.02154.

The estimated intracellular losses of arginine, guanidinoacetate, creatine and creatinine during hemodialysis were calculated as the difference between the total losses of arginine, guanidinoacetate, creatine and creatinine and the estimated extracellular losses of arginine, guanidinoacetate, creatine and creatinine: Estimated intracellular losses (μmol) = total losses (μmol) – estimated extracellular losses (μmol).

Higher estimated intracellular losses indicate a larger shift from the intra- to extracellular space during the hemodialysis session. Besides losses to the dialysate, there may also be losses in urine in patients with residual diuresis. To determine the degree to which urinary losses contribute the daily losses in patients with residual diuresis, we compared the urinary and the dialysate losses of arginine, guanidinoacetate, creatine and creatinine in patients with residual diuresis. The daily dialysate excretion rate of arginine, guanidinoacetate, creatine and creatinine are calculated as: Dialysate excretion rate (μmol/24-h) = (V_Dialysate_ * D_X_ * n) / 7). In this formula V_Dialysate_ refers to the total volume of dialysate (L), D_X_ refers to the measured concentration of arginine, guanidinoacetate, creatine and creatinine in the collected dialysate (μmol/L), and n refers to the number of dialysis sessions per week. The daily urinary excretion rate was calculated as: Urinary excretion rate (μmol/24-h) = (Urinary excretion_day 1_ + Urinary excretion_day 2_) / 2.

### Characteristics of the protein energy wasting phenotype

The cross-sectional outcomes low muscle mass, low protein intake, hypoalbuminemia, low body mass index and severe fatigue were used as characteristics for the PEW phenotype.

Muscle mass was assessed by calculating the combined creatinine excretion rate (CER):

Creatinine excretion rate (mmol/24-h) = ((V_Dialysate_ * D_Creatinine_ * n) / 7 + UCrE). In this formula V_Dialysate_ refers to the total volume of dialysate (L), D_Creatinine_ refers to measured creatinine concentration in the collected dialysate (mmol/L), n refers number of dialyses per week, and UCrE refers to 24-h urinary creatinine excretion (mmol/24-h), averaged from two 24-h urine collections. The creatinine excretion rate was converted to the skeletal muscle index by multiplying by the creatinine-equivalence (1 g of creatinine excretion per 24-h (8.84 mmol) = 22.73 kg of skeletal muscle mass) and dividing by height squared [29, 30]. A low muscle mass was defined as a SMI < 10.76 kg/m^2^ in males and < 6.76 kg/m^2^ in females [29, 31, 32].

Low protein intake was defined as a dietary protein intake < 0.8 g/kg/24-h [2]. The dietary protein intake was calculated based on the combined (dialysate and urinary) excretion rate of urea, according to the Maroni formula and indexed to body weight [33]:

Protein intake (g/kg/24-h) = (6.25 * (0.028 * CUER + 0.031 * BW) + UPE) / BW.

In this formula CUER refers to combined excretion rate of urea (mmol/24-h), BW refers to body weight postdialysis (kg), and UPE refers to the 24-h urine protein excretion (g/24-h), averaged from two 24-h urine collections. CUER was calculated as follows: CUER (in mmol/24-h) = (V_Dialysate_ * D_urea_ * n) / 7 + UUE. In this formula, V_Dialysate_ refers total volume of the spent dialysate (L), D_urea_ refers to the measured urea concentration in the collected dialysate (mmol/L), n refers to the number of hemodialysis sessions per week, and UUE refers to the 24-h urinary urea excretion (mmol/24-h), averaged from two 24-h urine collections. Hypoalbuminemia was defined as a plasma albumin concentration < 38 g/L [2]. Low body mass was defined as a body mass index < 23 kg/m^2^ [2]. Severe fatigue was defined as a subjective fatigue score of ≥ 35 on the checklist individual strength [23, 24]. An overview of all used formulas can be found in Additional file 1: Table S10.

### Statistical analysis

Data analyses and computations were performed with R version 3.6.1 software (http://cran.r-project.org/). A two-sided P < 0.05 was considered to indicate statistical significance. Data were presented as mean ± standard deviation for normally distributed data, median [interquartile range] for non-normally distributed data, and as numbers (percentages) for nominal data. Baseline characteristics, intradialytic plasma concentration changes, single hemodialysis removal and comparisons of urinary excretion rate and dialysate excretion rate were shown for all participants and stratified according to sex. Sex-stratified analyses were performed based on the known sex-based differences in creatine homeostasis [5, 34] as well as editorial statements calling for clinical trials and observational studies to report on sex-stratified results [35, 36]. The differences between males and females were tested using the independent sample t-test or the Mann–Whitney U test. Differences between pre- and postdialysis plasma concentrations, between extracellular removal and intracellular removal and between the urinary excretion rate and the dialysate excretion rate were tested using the paired sample t-test or the Wilcoxon signed-rank test. Correlations between predialysis plasma concentrations and the dialysate losses of arginine, guanidinoacetate, creatine and creatinine were assessed using the Pearson correlation coefficient. Associations of predialysis plasma concentrations of arginine, guanidinoacetate, creatine, and creatinine with low muscle mass, low protein intake, low plasma albumin, low BMI and severe fatigue were investigated using logistic regression analyses. Due to the small sample size, these analyses were not stratified according to sex, but performed with adjustment for sex. In addition, multivariable analyses were performed in which we additionally adjusted for the a priori selected potential confounders age, sex, body surface area, dialysis vintage and high-sensitivity C-reactive protein (hs-CRP). Due to a skewed distribution, plasma creatine concentrations were log_2_-transformed prior to logistic regression analyses. To determine whether the found associations were driven by patients with high plasma creatine concentrations, we performed sensitivity analyses after prior exclusion of patients with predialysis plasma creatine concentrations above the 95th percentile. In addition, we also performed sensitivity analyses in which we adjusted for the type of vascular access. Also, we also performed sensitivity analyses in which we adjusted for the hemodialysis efficacy (Kt/V). Furthermore, we performed sensitivity analyses in which we adjusted for the usage of erythropoietin-stimulating agents, vitamin D analogues and corticosteroids. Lastly, we performed sensitivity analyses in which we adjusted for the presence or absence of residual diuresis. To visualize the continuous associations of plasma creatine concentrations with low muscle mass, low protein intake, hypoalbuminemia and severe fatigue, plasma creatine concentration, as a continuous variable, was individually plotted against the probability of low muscle mass, low protein intake, hypoalbuminemia and severe fatigue using the *visreg* package in R. These analyses were adjusted for sex, age, body surface area, dialysis vintage and hs-CRP.

## Results

### Baseline characteristics

A total of 59 hemodialysis patients were included in the study, 37 (63%) of whom were male. Mean age at inclusion was 65 ± 15 years with a median hemodialysis vintage of 15 [6–41] months. Nearly all (95%) patients dialyzed thrice-weekly and most patients (81%) dialyzed four hours per session. A total of 32 (54%) patients had residual diuresis, with a mean urinary volume of 0.9 ± 0.6 L. Vascular access was a lower arm arteriovenous fistula in 13 (22%) patients, an upper arm arteriovenous fistula in 19 (32%) patients, a central venous line in 19 (32%) patients and an arteriovenous graft in 8 (14%) patients. The mean Kt/V was 1.4 ± 0.3 per hemodialysis session. Mean BMI was 25.5 ± 4.3 kg/m^2^. Median hs-CRP was 4.9 [1.7–13.5] mg/L and median IL-6 was 5.6 [3.2–13.0] pg/mL. Hypertension, cardiovascular disease, and diabetes mellitus were prevalent in 33 (58%), 25 (42%) and 15 (25%) of the patients, respectively. Erythropoietin-stimulating agents, vitamin D analogues, corticosteroids and androgens were used by 49 (83%), 38 (64%), 12 (20%) and 2 (3%) patients, respectively. Differences in baseline characteristics stratified by sex are shown in Table 1. Compared to males, females had lower body weight, height and BSA (all P < 0.05). Compared to males, females had a higher Kt/V (P = 0.01). Other baseline characteristics were comparable between males and females.

**Table 1:** Baseline characteristics of the hemodialysis patients

	Total cohort	Males	Females	P-value
Demographics				
Participants, n (%)	59 (100)	37 (63)	22 (37)	0.05
Age, years	65 ± 15	65 ± 14	64 ± 15	0.8
Race, n Caucasian (%)	52 (88)	31 (84)	21 (95)	0.4
Dialysis-related				
Dialysis vintage, months	15 [6–39]	15 [7–29]	16 [6–48]	0.7
Dialysis sessions, n (%)				
Two sessions per week	3 (5)	1 (3)	2 (9)	0.6
Three sessions per week	56 (95)	36 (97)	20 (91)	
Hours per dialysis, n (%)				
3 to 3.5 h	6 (10)	3 (8)	3 (14)	0.6
4 h	48 (81)	30 (81)	18 (82)	
4.5 to 5 h	5 (8)	4 (11)	1 (5)	
Dialysate volume, L	135 ± 27	140 ± 30	127 ± 23	0.08
Ultrafiltration volume, L	1.9 ± 0.9	1.9 ± 0.9	1.9 ± 0.8	0.8
Residual diuresis, n (%)	32 (54)	24 (65)	8 (36)	0.06
Urinary volume, L	0.9 ± 0.6	0.8 ± 0.6	1.0 ± 0.7	0.4
Kt/V	1.4 ± 0.3	1.3 ± 0.3	1.6 ± 0.3	0.01
Vascular access, n (%)				
Lower arm fistula	13 (22)	11 (30)	2 (9)	0.3
Upper arm fistula	19 (32)	11 (30)	8 (36)	
Central venous line	19 (32)	11 (30)	8 (36)	
Arteriovenous graft	8 (14)	4 (11)	4 (18)	
Body composition				
Weight predialysis, kg	80 ± 16	84 ± 17	74 ± 13	0.03
Weight postdialysis, kg	78 ± 16	82 ± 16	73 ± 13	0.04
Height, m	1.75 ± 0.09	1.80 ± 0.07	1.67 ± 0.05	< 0.001
BMI ^a^, kg/m^2^	25.5 ± 4.3	25.2 ± 4.4	26.1 ± 4.2	0.4
BSA ^a^, m^2^	1.94 ± 0.21	2.01 ± 0.20	1.81 ± 0.16	< 0.001
Inflammation				
Hs-CRP, mg/L	4.9 [1.7–13.5]	4.9 [1.6–12.0]	5.3 [2.0–17.5]	0.8
IL-6, pg/mL ^b^	5.6 [3.2–13.0]	6.4 [3.2–15.0]	5.2 [3.6–7.2]	0.5
Pre-existing disease				
Hypertension, n (%)	33 (58)	24 (65)	9 (41)	0.1
Cardiovascular disease, n (%)	25 (42)	16 (43)	9 (41)	0.9
Diabetes, n (%)	15 (25)	10 (27)	5 (23)	0.9
Medication				
Erythropoietin-stimulating agents, n (%)	49 (83)	29 (78)	20 (90)	0.4
Vitamin D analogues, n (%)	38 (64)	23 (62)	15 (68)	0.9
Corticosteroids, n (%)	12 (20)	6 (10)	6 (27)	0.5
Androgens, n (%)	2 (3)	2 (5)	0 (0)	0.7

^a^Calculated using postdialysis weight

^b^Data available in 39 participants

### Intradialytic changes in plasma concentrations.

Intradialytic changes in plasma concentrations of arginine, guanidinoacetate, creatine and creatinine are shown in Table 2. Plasma arginine concentrations decreased during dialysis from 77 ± 22 μmol/L to 60 ± 19 μmol/L (P < 0.001), corresponding to a proportional decrease of 18 ± 25%. No significant sex-based differences were found. During hemodialysis, plasma guanidinoacetate concentrations decreased from 1.8 ± 0.6 μmol/L to 1.0 ± 0.3 μmol/L (P < 0.001), corresponding to a proportional decrease of 40 ± 17%. No significant sex-based differences were found. Plasma creatine concentrations decreased from 26 [16–41] μmol/L to 21 [15–30] μmol/L (P < 0.001), corresponding to a proportional decrease of 13 ± 39%. Males had lower predialysis (19 [15–33] μmol/L vs 33 [24–56] μmol/L; P = 0.01) and postdialysis plasma creatine concentrations (20 [15–25] μmol/L vs 24 [15–37] μmol/L; P = 0.01), as compared to females. Additionally, in males, the proportional decrease in plasma creatine concentration was smaller (5 ± 43% vs 27 ± 26%; P = 0.02), as compared to females.

**Table 2:** Intradialytic changes of plasma concentrations of arginine, guanidinoacetate, creatine and creatinine

Metabolite	Total cohort	Males	Females	P-value
Arginine				
Predialysis concentration, μmol/L	77 ± 22^***^	80 ± 24^***^	72 ± 18^***^	0.2
Postdialysis concentration, μmol/L	60 ± 19^***^	63 ± 21^***^	55 ± 14^***^	0.08
Absolute decrease, μmol/L	17 ± 23^***^	17 ± 25^***^	16 ± 19^***^	0.9
Proportional decrease, %	18 ± 25^***^	17 ± 28^***^	20 ± 21^***^	0.7
Guanidinoacetate				
Predialysis concentration, μmol/L	1.8 ± 0.6^***^	1.9 ± 0.6^***^	1.6 ± 0.5	0.1
Postdialysis concentration, μmol/L	1.0 ± 0.3^***^	1.1 ± 0.3^***^	0.9 ± 0.4	0.07
Absolute decrease, μmol/L	0.8 ± 0.4^***^	0.8 ± 0.5^***^	0.7 ± 0.4^***^	0.4
Proportional decrease, %	40 ± 17^***^	38 ± 17^***^	43 ± 15^***^	0.3
Creatine				
Predialysis concentration, μmol/L	26 [16–41] ^***^	19 [15–33] ^*^	33 [24–56] ^***^	0.01
Postdialysis concentration, μmol/L	21 [15–30] ^***^	20 [15–25] ^*^	24 [15–37] ^***^	0.01
Absolute decrease, μmol/L	5 [1–14] ^***^	3 [0–8] ^*^	10 [3–18] ^***^	0.02
Proportional decrease, %	13 ± 39^*^	5 ± 43	27 ± 26^***^	0.02
Creatinine				
Predialysis concentration, μmol/L	689 ± 207^***^	706 ± 224^***^	660 ± 175^***^	0.4
Postdialysis concentration, μmol/L	257 ± 92^***^	274 ± 97^***^	228 ± 78^***^	0.07
Absolute decrease, μmol/L	432 ± 146^***^	432 ± 156^***^	432 ± 130^***^	0.9
Proportional decrease, %	63 ± 8^***^	61 ± 9^***^	65 ± 7^***^	0.05

Differences between males and females are tested using an independent sample t-test. Differences between predialysis and postdialysis plasma concentrations are tested using paired sample t-test or the paired samples Wilcoxon test. Absolute decrease and proportional decrease are test against no decrease by t-test or Wilcoxon test. Statistical significance is indicated with asterixis (^*^ P < 0.05; ^**^ P < 0.01; ^***^ P < 0.001)

Plasma creatinine concentrations decreased from 689 ± 207 μmol/L to 257 ± 92 μmol/L (P < 0.001), corresponding to a proportional decrease of 63 ± 8%. In contrast to creatine, predialysis plasma concentrations of creatinine (706 ± 224 μmol/L vs 660 ± 175 μmol/L; P = 0.4) and postdialysis plasma concentrations of creatinine (274 ± 97 μmol/L vs 228 ± 78; P = 0.07) were numerically higher in males, as compared to females, albeit not significant at the 5% level.

### Losses during hemodialysis

The losses of arginine, guanidinoacetate, creatine and creatinine during a hemodialysis session are shown in Table 3. During hemodialysis, the total loss of arginine was 1939 ± 871 μmol, of which an estimated amount of 287 ± 344 μmol (15%) was lost from the extracellular compartment and an estimated amount of 1655 ± 859 μmol (85%) was lost from the intracellular compartment (P < 0.001). No significant sex-based differences were found for arginine removal. The total loss of guanidinoacetate was 37 ± 20 μmol, of which an estimated amount of 12 ± 7 μmol (32%) was lost from the extracellular compartment and an estimated amount of 25 ± 19 μmol (68%) was lost from the intracellular compartment (P < 0.001). The total losses of guanidinoacetate were larger in males than in females (41 ± 20 μmol vs 31 ± 18 μmol; P = 0.05). The total loss of creatine was 719 [399–1070] μmol, of which an estimated amount of 79 [19–196] μmol (18%) was lost from the extracellular compartment and an estimated amount of 612 [365–902] μmol (82%) was lost from the intracellular compartment (P < 0.001).

**Table 3:** Comparison of the dialysate losses of arginine, guanidinoacetate, creatine and creatinine per hemodialysis session

Metabolite		Total cohort	Males	Females	P-value
Arginine					
Total loss, μmol		1939 ± 871	2053 ± 921	1748 ± 760	0.2
Estimated extracellular loss, μmol		287 ± 344^***^	318 ± 390^***^	235 ± 248^***^	0.3
Estimated intracellular loss, μmol		1655 ± 859^***^	1747 ± 922^***^	1497 ± 733^***^	0.3
Guanidinoacetate					
Total loss, μmol		37 ± 20	41 ± 20	31 ± 18	0.05
Estimated extracellular loss, μmol		12 ± 7^***^	13 ± 8^***^	10 ± 5^**^	0.08
Estimated intracellular loss, μmol		25 ± 19^***^	28 ± 20^***^	21 ± 16^**^	0.2
Creatine					
Total loss, μmol		719 [399–1070]	655 [397–1015]	867 [464–1363]	0.2
Estimated extracellular loss, μmol		79 [19–196] ^***^	58 [5–144] ^***^	147 [42–231] ^***^	0.04
Estimated intracellular loss, μmol		612 [365–902] ^***^	545 [365–871] ^***^	697 [413–1052] ^***^	0.4
Creatinine					
Total loss, mmol		15.5 ± 8.4	17.5 ± 9.6	12.0 ± 3.9	0.003
Estimated extracellular loss, mmol		6.8 ± 2.5^*^	7.2 ± 2.7^*^	6.2 ± 1.9	0.2
Estimated intracellular loss, mmol		8.7 ± 7.1^*^	10.4 ± 8.4^*^	5.8 ± 2.2	0.003

ECV is calculated according to the formula by Abraham et al. [27]

Differences between males and females are tested using an independent sample t-test or Mann–Whitney U test. Differences between extracellular and intracellular removal are tested using paired sample t-test or the paired samples Wilcoxon test, and statistical significance is indicated with asterixis (^*^ P < 0.05; ^**^ P < 0.01; ^***^ P < 0.001)

The total loss of creatinine was 15.5 ± 8.4 mmol, of which an estimated amount of 6.8 ± 2.5 mmol (44%) was lost from the extracellular compartment and an estimated amount of 8.7 ± 7.1 mmol (56%) was lost from the intracellular compartment (P < 0.05). The total losses of creatinine were larger in males than in females (17.5 ± 9.6 mmol vs 12.0 ± 3.9 mmol; P = 0.003).

**Fig. 1:**
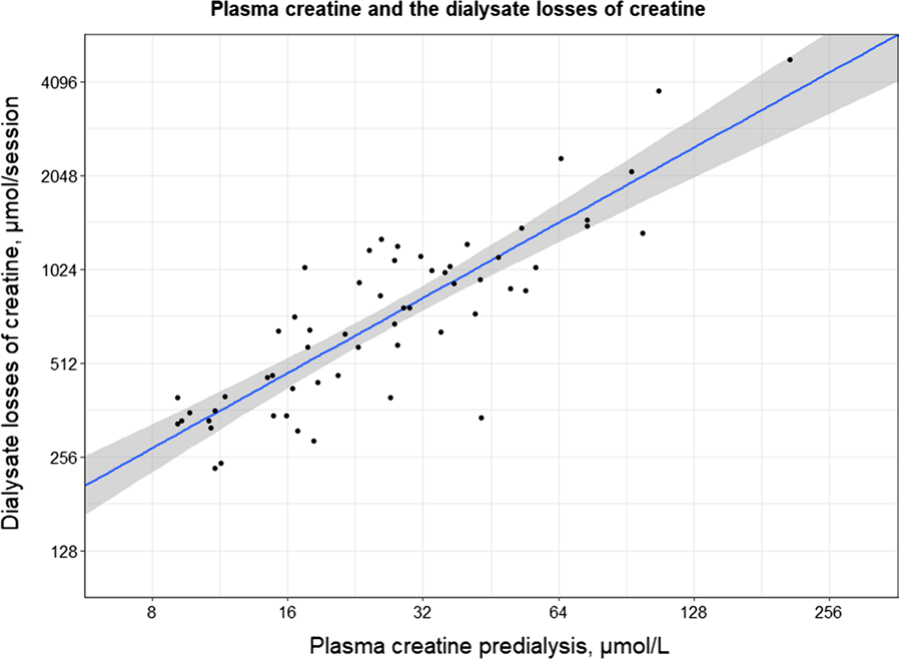
Scatterplot of plasma creatine predialysis and the dialysate losses of creatine. Pearson correlation coefficient = 0.90 (P < 0.001). Blue line represents the association between the dialysate losses of creatine and the plasma creatine concentration, and the shaded area represents the 95% confidence interval

Correlation analyses of predialysis plasma values and dialysate losses are shown in Additional file 1: Table S2. Predialysis plasma concentrations were positively correlated with dialysate losses for arginine (r = 0.51; P < 0.001), guanidinoacetate (r = 0.54; P < 0.001), creatine (r = 0.90; P < 0.001) and creatinine (r = 0.60; P < 0.001). A graphical representation of the correlation between predialysis plasma creatine and the dialysate losses of creatine is shown in Fig. 1, showing that higher predialysis plasma creatine concentrations are associated with larger dialysate losses of creatine.

Since hemodialysis patients with residual diuresis will have losses into urine in addition to losses to dialysate, we compared the urinary losses to the dialysate losses. An overview of the daily urinary and dialysate excretion rates of arginine, guanidinoacetate, creatine and creatinine is shown in Additional file 1: Table 3. In patients with residual diuresis, urinary excretion contributes 2.8% to the total daily excretion rate of arginine, 42% to the total daily excretion rate of guanidinoacetate, 6.0% to the total daily excretion rate of creatine and 38% to the total daily excretion rate of creatinine. No sex-based differences were found for the proportion that daily urinary excretion rate contributes to the total daily excretion rate.

### Characteristics of the protein energy wasting phenotype

Associations of predialysis plasma concentrations of arginine, guanidinoacetate, creatine and creatinine with low muscle mass, low protein intake, hypoalbuminemia, low BMI, and severe fatigue are shown in Table 4. In the sex-adjusted models, higher plasma arginine was inversely associated with low protein intake (OR per standard deviation decrease: 1.94 [1.08–3.94]; P = 0.04) and hypoalbuminemia (OR: 2.50 [1.35–5.33]; P = 0.007). In multivariable analyses, the association with low protein intake was lost, while the association with hypoalbuminemia remained materially unchanged. No associations were found between plasma arginine and low muscle mass, low BMI, or severe fatigue (all P > 0.05). For plasma guanidinoacetate, no associations were found with low muscle mass, low protein intake, hypoalbuminemia, low BMI, or severe fatigue (all P > 0.05).

**Table 4:** Associations of predialysis plasma concentrations of arginine, guanidinoacetate, creatine and creatinine with characteristics of the protein energy wasting phenotype

	Sex-adjusted analyses		Multivariable analyses	
	OR (95% CI)	P value	OR (95% CI)	P value
Plasma arginine (per 1-SD decrease)				
Low muscle mass	1.65 [0.84–3.89]	0.2	1.60 [0.74–4.27]	0.3
Low protein intake	1.94 [1.08–3.94]	**0.04**	1.91 [1.01–4.16]	0.07
Hypoalbuminemia	2.50 [1.35–5.33]	**0.007**	2.87 [1.39–7.06]	**0.009**
Low BMI	1.06 [0.59–1.86]	0.8	0.91 [0.32–2.89]	0.9
Severe fatigue	1.14 [0.63–2.08]	0.7	1.04 [0.50–2.15]	0.9
Plasma guanidinoacetate (per 1-SD decrease)				
Low muscle mass	1.08 [0.59–2.19]	0.8	0.94 [0.48–2.08]	0.9
Low protein intake	1.55 [0.89–2.94]	0.2	1.11 [0.60–2.24]	0.7
Hypoalbuminemia	1.45 [0.82–2.65]	0.2	1.35 [0.71–2.67]	0.3
Low BMI	1.17 [0.66–2.08]	0.6	1.61 [0.70–3.77]	0.2
Severe fatigue	1.20 [0.69–2.15]	0.5	0.96 [0.49–1.84]	0.9
Plasma creatine (per halving)				
Low muscle mass	2.00 [1.05–4.14]	**0.04**	2.32 [1.10–5.69]	**0.04**
Low protein intake	2.13 [1.17–4.27]	**0.02**	2.37 [1.20–5.41]	**0.02**
Hypoalbuminemia	3.13 [1.46–8.02]	**0.008**	6.20 [2.16–24.8]	**0.003**
Low BMI	0.74 [0.40–1.35]	0.3	0.51 [0.18–1.28]	0.2
Severe fatigue	3.20 [1.52–8.05]	**0.006**	3.74 [1.55–11.8]	**0.009**
Plasma creatinine (per 1-SD decrease)				
Low muscle mass	6.66 [2.14–28.6]	**0.003**	5.67 [1.59–37.3]	**0.03**
Low protein intake	1.35 [0.80–2.43]	0.3	3.15 [0.82–3.29]	0.2
Hypoalbuminemia	2.51 [1.35–5.41]	**0.006**	2.50 [1.25–5.72]	**0.02**
Low BMI	1.80 [1.02–3.45]	**0.05**	2.58 [0.94–9.66]	0.10
Severe fatigue	1.09 [0.62–1.90]	0.76	1.65 [0.80–3.51]	0.18

Plasma refers to plasma values predialysis. Analyses of plasma arginine, guanidinoacetate and creatinine are performed on scaled data, with the odds ratio presented per standard deviation decrease of the biomarker. Analyses of plasma creatine are performed on log_2_ transformed data, with the odds ratio presented per halving of the biomarker

Low muscle mass is defined as a muscle mass < 10.76 kg/m^2^ in males < 6.76 kg/m^2^ in females. Low protein intake is defined as a protein intake < 0.8 g/kg/24-h. Low BMI is defined as a BMI < 23 kg/m2. Hypoalbuminemia is defined as a serum albumin < 38 g/L. Severe fatigue is defined as a subjective fatigue score ≥ 35. Multivariable analyses are adjusted for sex, age, body surface area, dialysis vintage and hs-CRP

**Fig. 2:**
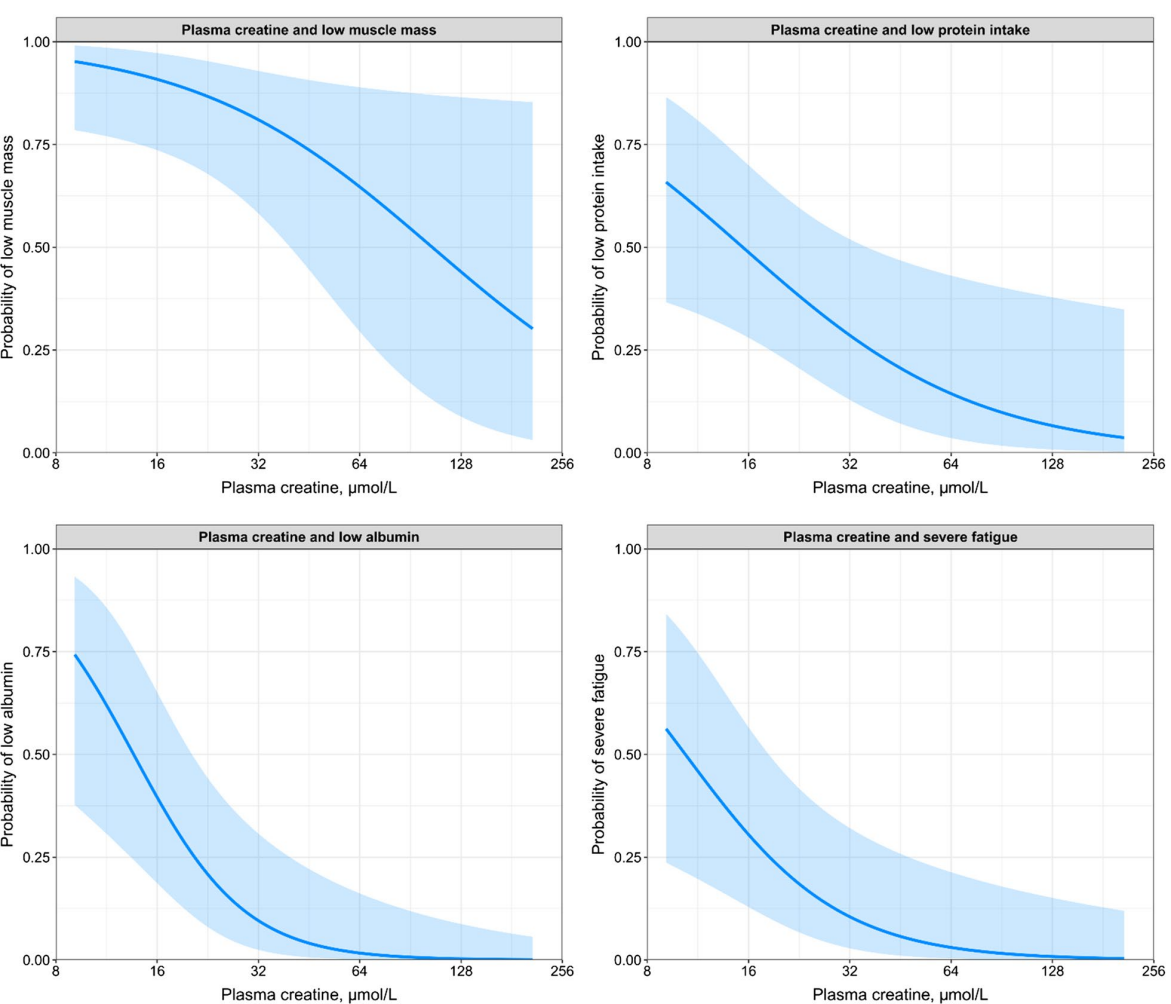
Graphical representation of the associations between plasma creatine and the probability of low muscle mass, low protein intake, hypoalbuminemia, and severe fatigue. Analyses are adjusted for sex, age, body surface area, dialysis vintage and hs-CRP

In sex-adjusted models, lower plasma creatine was associated with higher odds of low muscle mass (OR per halving of plasma creatine: 2.00 [1.05–4.14]; P = 0.04), low protein intake (OR: 2.13 [1.17–4.27]; P = 0.02), hypoalbuminemia (OR: 3.13 [1.46–8.02]; P = 0.008) and severe fatigue (OR: 3.20 [1.52–8.05]; P = 0.006). After adjustment for potential confounders, these associations remained materially unchanged. No associations were found with low BMI. A graphical representation of the relationship between plasma creatine and low muscle mass, low protein intake, hypoalbuminemia and severe fatigue is shown in Fig. 2.

In sex-adjusted models, lower plasma creatinine was associated with low muscle mass (OR per standard deviation decrease: 6.66 [2.21–28.6]; P < 0.001) and hypoalbuminemia (OR: 2.51 [1.35–5.41]; P = 0.02). After adjustment for potential confounders, these associations remained materially unchanged. No associations were found with low protein intake, low BMI and severe fatigue.

### Sensitivity analyses

To determine whether the associations of plasma creatine are driven by the patients with the highest creatine concentrations, we performed sensitivity analyses in which patients with predialysis plasma creatine values above the 95th percentile were excluded in Additional file 1: Table S4. In these analyses, the association of plasma creatine with low muscle mass was no longer significant (P = 0.20). However, the association between plasma creatine and low protein intake, hypoalbuminemia and severe fatigue remained significant and materially unchanged.

To investigate whether the associations of plasma creatine with low muscle mass, low protein intake, hypoalbuminemia and severe fatigue could be the consequence of differences in vascular access type, we performed sensitivity analyses in which we adjusted for the type of vascular access (Additional file 1: Table S5). The associations remained materially unchanged after adjustment for the type of vascular access. To investigate whether the associations of plasma creatine with low muscle mass, low protein intake, hypoalbuminemia and severe fatigue could be the consequence of differences in hemodialysis efficacy, we performed sensitivity analyses in which we adjusted for the Kt/V values (Additional file 1: Table S6). The associations remained materially unchanged after adjustment for the Kt/V. To investigate whether the associations of plasma creatine with low muscle mass, low protein intake, hypoalbuminemia and severe fatigue could be the consequence of differences in medication usage, we performed sensitivity analyses in which we adjusted for the usage of erythropoietin-stimulating agents, vitamin D analogues and corticosteroids (Additional file 1: Table S7). The associations remained materially unchanged after adjustment for these medications. To investigate whether the associations of plasma creatine with low muscle mass, low protein intake, hypoalbuminemia and severe fatigue could be the consequence of differences in residual diuresis, we performed sensitivity analyses in which we adjusted the associations for presence or absence of residual diuresis (Additional file 1: Table S8). The associations remained materially unchanged after adjustment for the presence or absence of residual diuresis. Associations of the dialysate losses of arginine, guanidinoacetate, creatine and creatinine with low muscle mass, low protein intake, hypoalbuminemia, low BMI, and fatigue are shown in Additional file 1: Table S9**.** For the dialysate losses of arginine and guanidinoacetate no associations were found with low muscle mass, low protein intake, hypoalbuminemia, low BMI, or fatigue. Lower dialysate losses of creatine were associated with higher odds of low muscle mass (OR per halving: 2.61 [1.28–6.31]; P = 0.02), hypoalbuminemia (OR: 4.77 [2.03–13.8]; P = 0.001) and severe fatigue (OR: 2.80 [1.34–6.65]; P = 0.01). After adjustment for potential confounders, these associations remained materially unchanged. No significant associations were found with low protein intake and low BMI.

## Discussion

To our knowledge, this is the first study to comprehensively investigate creatine homeostasis in hemodialysis patients. Arginine, guanidinoacetate, creatine and creatinine all demonstrated an intradialytic decrease in plasma concentrations. The proportional decrease was greatest for creatinine and lowest for creatine. No sex-differences were found for arginine, guanidinoacetate and creatinine. By contrast, males had lower pre- and postdialysis plasma creatine concentrations and had a lower proportional intradialytic decrease in plasma concentrations compared to females. In addition, we quantified the intradialytic losses and demonstrated that the majority of creatine is removed from the intracellular compartment, thereby implicating a large intra- to extracellular shift of creatine during a hemodialysis session. For patients with residual diuresis we demonstrated that the intradialytic losses of arginine, guanidinoacetate, creatine and creatinine to the dialysis fluid are much larger than the interdialytic urinary losses. Lastly, we demonstrated that lower plasma creatine concentrations are associated with higher odds of low muscle mass, low protein intake, hypoalbuminemia, and severe fatigue. These findings were independent from potential confounders, including plasma levels of hs-CRP as a reflection of systemic inflammation.

Creatine and the energy-charged phosphocreatine play a crucial bioenergetic role in adenosine triphosphate turnover and are especially important as a cellular energy buffer and in energy transport, especially in tissues with high and fluctuating energetic demands, such as skeletal muscles and the brain [37, 38]. Creatine is therefore an essential metabolite for normal cell and body functioning, from embryonic life to adulthood [39]. The importance of creatine is highlighted by the genetic creatine deficiency syndromes, encompassing mutations in the genes of AGAT, GAMT or the gene coding for the creatine transporter (SLC6A8), where patients develop neuromuscular and neurological symptoms, including mental retardation, autism, and epilepsy [40]. In omnivorous humans, creatine is synthesized endogenously, but also ingested through the diet [7]. After being released into the circulation, creatine is transported into tissues by the Creatine Transporter 1 (CrT1), encoded by the SLC6A8 gene, located on the X-chromosome [41]. Throughout the years, several sex-based differences in creatine homeostasis have been identified, e.g. the overall creatine synthesis rate has been found to be lower in females than in males [5], which is also reflected by lower serum guanidinoacetate concentrations in females than in males [34]. A study in 66 healthy individuals found lower serum guanidinoacetate concentrations in females (2.0 ± 0.57 μmol/L) than in males (2.6 ± 0.52 μmol/L) [42]. Compared to the healthy individuals of the aforementioned study, the peak (predialysis) plasma concentrations in the hemodialysis patients of the current study were 27% and 20% lower in males and females, respectively. These findings support previous hypotheses that the first step of endogenous creatine synthesis is impaired in patients with end-stage kidney disease [8, 15]. Especially since generally plasma concentrations of small water-soluble molecules tend to increase with decreasing kidney function [43]. Since the conversion of AGAT is considered the rate limiting step in creatine synthesis [7–14], it follows that a reduction in AGAT activity in end-stage kidney disease also leads to reduced creatine concentrations, compatible with our findings. It is worth noting that the concentrations of creatine in healthy individuals of the study by Marescau et al. [42] were nearly twice times higher in females, compared with males. Similarly, we also found higher plasma creatine concentrations in females as compared with males, both in pre- and postdialysis samples. Although the exact mechanism underlying this sex-based difference is not known, it has been reported before [44], and the same phenomenon is also seen in biopsies of the vastus lateralis muscle where females had 10% higher intracellular creatine concentrations compared to males [45]. Our analysis of the intradialytic changes in plasma concentrations demonstrated that arginine, guanidinoacetate, creatine and creatinine all significantly decreased during dialysis, in line with small molecules being removed to the dialysate. Interestingly, the proportional decrease in plasma concentrations was largest (63%) for creatinine and smallest for creatine (13%), implicating that plasma creatine concentrations are more prone to strict regulation. To elucidate losses to the dialysate, we quantified the losses to the dialysate during hemodialysis by collecting the entirety of the spent dialysate. In agreement with the lower plasma concentrations of guanidinoacetate compared to creatine, we also found less removal of guanidinoacetate as compared to creatine. By estimating the relative losses from the extracellular and intracellular compartments, we demonstrated that most creatine was lost from the intracellular compartment, indicative of a large intradialytic intra- to extracellular shift. A possibility is that plasma creatine is strictly regulated and that an intra- to extracellular shift is one of the mechanisms preventing too low plasma creatine concentrations. This hypothesis of a large intra- to extracellular shift during hemodialysis is congruent with previous imaging studies obtained by non-invasive in vivo 31P-NMR demonstrating lower phosphocreatine concentrations in the hearts of patients on either hemodialysis treatment [46] or peritoneal dialysis treatment [47]. Additionally, lower phosphocreatine concentrations have also been measured in skeletal muscles of hemodialysis patients [48]. Combined, these findings indicate a wash-out of creatine from these organs during hemodialysis treatment.

Apart from losses to the dialysate, there may also be losses of creatine to the urine in patients with residual diuresis. Yet, in our supplementary analyses we demonstrated that urinary losses only contribute a minor 6.0% to the overall daily losses of creatine in hemodialysis patients.

Protein energy wasting is a major threat for patients on hemodialysis, as it is the strongest risk factor for morbidity and mortality [1, 3]. Clinically, the diagnostic criteria have been set up across several domains, including low muscle mass, low protein intake, hypoalbuminemia and low body weight. A relatively simple, non-invasive and very reliable method to estimate muscle mass is to measure the creatinine excretion rate (CER). Creatinine is produced at a constant rate, depending on the quantity of muscle mass [7, 49]. Therefore, CER is an established method to study total body muscle mass in both healthy populations and a variety of patient populations, including patients with chronic kidney disease [15, 50–54]. An added benefit of CER over other techniques, such as dual-energy x-ray absorptiometry, is that CER by its biochemical nature is insensitive to hydration status, intramuscular fat, and edema and thereby provides a direct reflection of muscle mass [55, 56]. Lower plasma values of creatine, but not arginine and guanidinoacetate, were associated with higher odds for a low muscle mass, indicating that low plasma creatine concentrations may increase the risk of low muscle mass. Similarly, we found that lower plasma creatine concentrations were associated with higher odds of low protein intake and hypoalbuminemia. In contrast to low muscle mass, these associations remained materially unchanged after excluding the patients with highest plasma creatine concentrations. To assess protein intake, we used a biomarker-based method to assess dietary protein intake, thereby avoiding potential biases of classic dietary assessments, including under- and overreporting, changes in diet due to self-reflections, errors in portion size estimates, and socially desirable answers [15, 57, 58]. Additionally, we also found that higher plasma creatine values are associated with a reduced odds of severe fatigue. Although fatigue is not a part of the diagnostic criteria for PEW, it is an often-occurring symptom in hemodialysis patients, especially in those with PEW [59, 60]. We purposely included fatigue, as fatigue is an often under-recognized and under-treated symptom and is one of the most debilitating symptoms experienced during hemodialysis treatment from a patient’s perspective. This is well-illustrated by a study demonstrating that 94% of the patients would accept more intense hemodialysis if it would increase their energy level, whereas merely 19% would accept this for an increase in longevity by 3 years [61].

The lower plasma values of guanidinoacetate and creatine compared to previous reports in healthy individuals and the found associations of plasma creatine with characteristics of PEW points toward a potential role for creatine supplementation in hemodialysis patients. Although the absolute decrease in plasma creatine from 26 to 21 µmol/L seems rather small, the values should be interpreted in the context of the fact that creatine, like creatinine, is a small, water-soluble molecule, of which one would anticipate plasma concentrations to be high rather than low in the case of renal failure. Although reported variation in fasting plasma creatine concentrations is high [62, 63], the median fasting plasma concentration of 26 µmol/L observed in the hemodialysis patients in the current report is appreciably lower than reported mean values of 40.5 µmol/L in 6 healthy subjects in a creatine supplementation study and 36.7 µmol/L in 4,735 subjects of the general population [62, 63]. Interestingly, in the latter study, there was a significant inverse association between plasma creatine and plasma creatinine concentrations, which is in line with the low plasma creatine concentration that we observed in the current study, and signifies the likelihood of an important role of the kidney in endogenous creatine synthesis, which may be virtually absent in the context of dialysis-dependent chronic kidney disease [5, 7, 63]. Since hemodialysis is known to induce a generalized proteolytic response, particularly stimulating proteolysis from muscles [64], the falls in circulating concentrations of amino acids are lower and actual losses into the dialysate higher than one would expect in the absence of a proteolytic response [65]. Our data suggest that a similar scenario applies to creatine, with a relatively limited decrease in circulating concentrations and relatively large losses during hemodialysis. Creatine, other than amino acids, is important to be separately considered, because of its particular role in energy metabolism and bodily functions, including performance and cognitive functioning [4]. The hypothesis that dialysis treatment leads to lower intracellular creatine concentrations has also been demonstrated using imaging studies obtained by non-invasive in vivo 31P-NMR demonstrating lower phosphocreatine concentrations in the hearts of patients on either hemodialysis treatment [46] or peritoneal dialysis treatment [47].

Creatine supplementation has been studied extensively in non-CKD settings, where it has been shown that creatine supplementation together with moderate resistance training will counteract loss of muscle mass, caused by either age-related sarcopenia or due to immobilization (recently reviewed in [66]). Furthermore, evidence indicates that creatine supplementation improves brain health, improves cognition, and is effective in alleviating brain ischemia and hypoxia (recently reviewed in [67]). Furthermore, the daily average intake of creatine via meat and fish by the general population of the U.S.A. is 1.38 g of creatine per day [68]. The same study demonstrated that 42.8% of the participants had an average intake below the recommended level of 1 g of creatine per day [68]. It is very likely that this percentage would be even higher if hemodialysis patients were studied, for they are generally advised to consume less meat and fish compared to the general population [69, 70] and thus they are likely to be even more creatine deficient. Nonetheless, to date, only a few creatine supplementation trials have been conducted in hemodialysis patients. Nearly 20 years ago, Chang et al. [71] performed a small-scale randomized double-blind placebo-controlled trial of creatine supplementation to evaluate the potential of creatine to alleviate hemodialysis-associated muscle cramps in 10 hemodialysis patients. After 4 weeks of creatine supplementation, patients demonstrated a 60% reduction in muscle cramps, while no difference was found in the placebo group. After the wash-out period, the frequency of muscle cramps returned to the previous level [71]. More recently, Marini et al. (2019) performed a randomized double-blind placebo-controlled trial to evaluate whether oral creatine supplementation could attenuate loss of muscle mass and could reduce the malnutrition-inflammation score in 28 hemodialysis patients [72]. Compared to the placebo arm, the creatine treated arm demonstrated a significant increase in lean body mass after 4 weeks supplementation (effect size = 0.832 kg; P = 0.03). Additionally, the creatine treated arm demonstrated a sizable reduction of the malnutrition-inflammation score (effect size = 0.964; P = 0.01). In the latter study, after a loading phase with four times daily five grams of oral creatine for one week, the maintenance dosage was five grams of creatine per day. Besides potential problems with compliance, creatine also requires a large volume of water to be dissolved, which may negatively influence the fluid balance in patients already on a fluid restriction. In future studies, this may be circumvented by using intradialytic creatine supplementation [8], which also prevents the unopposed removal of creatine by hemodialysis treatment.

The strengths of this study are that we collected the entire dialysate production during the hemodialysis session instead of taking several samples, thereby increasing the accuracy of our assessments. In addition, we used a biomarker-based method to assess dietary protein intake, thereby avoiding potential biases of classic dietary assessments. However, we acknowledge that our study has limitations, primarily the relatively small sample size of this study. Our current study was too small to allow for analyses stratified for e.g. presence or absence of residual diuresis. Future larger studies are required to allow for investigation whether associations of plasma creatine with low muscle mass, low protein intake, hypoalbuminemia and severe fatigue differ in different strata of patients, e.g. strata of patients with or without residual diuresis. Additionally, we were unable to perform repeated measurements over time, to evaluate creatine homeostasis over a longer period of chronic hemodialysis treatment and to evaluate the associations between creatine status and loss of muscle mass and body weight over a prolonged time. Lastly, statistical significance generally does not necessarily indicate a biologically relevant effect and the possibility of residual confounding remains.

In conclusion, we investigated pre- and postdialysis plasma concentrations of arginine, guanidinoacetate, creatine and creatinine. Compared to available literature, plasma concentrations of guanidinoacetate and creatine were lower than in healthy individuals. In addition, we demonstrated that arginine, guanidinoacetate, creatine and creatinine are removed by hemodialysis, with a large intradialytic shift of creatine from the intra- to the extracellular compartment. Lastly, we demonstrate that lower plasma creatine concentrations are associated with higher odds of low muscle mass, low protein intake, hypoalbuminemia, and severe fatigue. Future research is required to determine whether creatine supplementation can help to prevent or treat protein energy wasting and fatigue.

## Supplementary Information

**Additional file 1. ** Additional Tables S1–S10.

## Data Availability

Data described in the manuscript, code book, and analytic code will be made available upon request of the editor.
