# Supplementary material for: Creatine homeostasis and protein energy wasting in hemodialysis patients
Source: J Transl Med. 2021 Mar 20;19:115. doi: 10.1186/s12967-021-02780-y (PMC7981955; doi:10.1186/s12967-021-02780-y)
Supplement: Supplementary file 1 — Additional file 1. Additional Tables S1–S10. [file 12967_2021_2780_MOESM1_ESM.docx]

**Additional data of:**

**Creatine Homeostasis and Protein Energy Wasting in Hemodialysis Patients**

Adrian Post^1,^*, Joëlle C. Schutten^1^, Daan Kremer^1^, Yvonne van der Veen^1^, Dion Groothof^1^, Camilo G. Sotomayor^1^, Christa A. Koops^2^, Pim de Blaauw^2^, Ido P. Kema^2^, Ralf Westerhuis^3^, Theo Wallimann^4^, M. Rebecca Heiner-Fokkema^2^, Stephan J.L. Bakker^1^ and Casper F.M. Franssen^1^

^1^ Department of Internal Medicine, University of Groningen, University Medical Center Groningen, 9713 GZ Groningen, The Netherlands

^2^ Department of Laboratory Medicine, University of Groningen, University Medical Center Groningen, Groningen, the Netherlands

^3^ Dialysis Center Groningen, 9713 GZ Groningen, The Netherlands

^4^ Department of Biology, ETH Zurich, Zurich, Switzerland.

***** Correspondence: a.post01@umcg.nl; Tel.: +31-649-653-442

**Email addresses according to author list:**

a.post01@umcg.nl; j.c.schutten@umcg.nl; d.kremer@umcg.nl; y.van.der.veen@umcg.nl; d.groothof@umcg.nl; c.g.sotomayor.campos@umcg.nl; c.a.koops@umcg.nl; p.de.blaauw@umcg.nl; i.p.kema@umcg.nl; r.westerhuis@dcg.nl; theo.wallimann@cell.biol.ethz.ch; m.r.heiner@umcg.nl; s.j.l.bakker@umcg.nl; c.f.m.franssen@umcg.nl.

**Data Share Statement**Data described in the manuscript, code book, and analytic code will be made available upon request of the editor.

**Corresponding author**

Adrian Post

Department of Internal Medicine, Division of Nephrology

University Medical Center Groningen

9700 RB Groningen, the Netherlands

Phone: +31649653442, Email: a.post01@umcg.nl

| **Table S1.** Comparison of the removal of arginine, guanidinoacetate and creatine during one hemodialysis session. | | | | | |
| --- | --- | --- | --- | --- | --- |
| **Metabolite** | | **Total cohort** | **Males** | **Females** | **P-value** |
| **Arginine** | |  |  |  |  |
|  | Total loss, μmol | 1939 ± 871 | 2053 ± 921 | 1748 ± 760 | 0.2 |
|  | Estimated extracellular loss, μmol | 285 ± 338^***^ | 316 ± 383^***^ | 232 ± 241^***^ | 0.3 |
|  | Estimated intracellular loss, μmol | 1657 ± 855^***^ | 1748 ± 916^***^ | 1500 ± 733^***^ | 0.3 |
| **Guanidinoacetate** | |  |  |  |  |
|  | Total loss, μmol | 37 ± 20 | 41 ± 20 | 31 ± 18 | 0.05 |
|  | Estimated extracellular loss, μmol | 12 ± 7^***^ | 13 ± 8^***^ | 10 ± 5^**^ | 0.08 |
|  | Estimated intracellular loss, μmol | 26 ± 19^***^ | 28 ± 20^***^ | 21 ± 16^**^ | 0.2 |
| **Creatine** | |  |  |  |  |
|  | Total loss, μmol | 719 [399 – 1070] | 655 [397 – 1015] | 867 [464 – 1363] | 0.2 |
|  | Estimated extracellular loss, μmol | 78 [19 – 186] | 57 [6 – 142] | 149 [42 – 221] | 0.04 |
|  | Estimated intracellular loss, μmol | 612 [372 – 902] | 551 [369 – 874] | 638 [413 – 1051] | 0.4 |
| **Creatinine** | |  |  |  |  |
|  | Total loss, mmol | 15.5 ± 8.4 | 17.5 ± 9.6 | 12.0 ± 3.9 | 0.003 |
|  | Estimated extracellular loss, mmol | 6.7 ± 2.5^**^ | 7.1 ± 2.7^***^ | 6.1 ± 2.0 | 0.2 |
|  | Estimated intracellular loss, mmol | 8.8 ± 7.1^**^ | 10.6 ± 8.4^***^ | 5.9 ± 2.2 | 0.003 |
| ECV is calculated according to the formula by Bird et al: ECV = Weight ^0.6469^ * Height ^0.7236^ * 0.02154.  Differences between males and females are tested using an independent sample t-test or Mann-Whitney U test. Differences between extracellular and intracellular removal are tested using paired sample t-test or the paired samples Wilcoxon test, and statistical significance is indicated with asterixis (^*^ P<0.05; ^**^ P<0.01; ^***^ P<0.001). | | | | | |

| **Table S2.** Correlations between predialysis plasma concentrations of arginine, guanidinoacetate and creatine and dialysate losses of arginine, guanidinoacetate, creatine and creatinine. | | | | | | |
| --- | --- | --- | --- | --- | --- | --- |
| **Metabolite** | **Total cohort** | | **Males** | | **Females** | |
|  | **Pearson's r** | **P-value** | **Pearson's r** | **P-value** | **Pearson's r** | **P-value** |
| Arginine | 0.51 | <0.001 | 0.49 | 0.002 | 0.51 | 0.01 |
| Guanidinoacetate | 0.54 | <0.001 | 0.47 | 0.003 | 0.62 | 0.002 |
| Creatine | 0.90 | <0.001 | 0.82 | <0.001 | 0.95 | <0.001 |
| Creatinine | 0.60 | <0.001 | 0.58 | <0.001 | 0.82 | <0.001 |
|  | | | | | | |

| **Table S3.** Daily urinary and dialysate excretion rates of arginine, guanidinoacetate, creatine and creatinine in 32 patients with residual diuresis. | | | | | |
| --- | --- | --- | --- | --- | --- |
| **Metabolite** | | **Total cohort** | **Males** | **Females** | **P-value** |
| **Arginine** | |  |  |  |  |
|  | Urinary excretion rate (μmol/24-h) | 14.1 ± 18.9^***^ | 12.3 ± 11.9^***^ | 18.8 ± 31.1^***^ | 0.4 |
|  | Dialysate excretion rate (μmol/24-h) | 745 ± 344^***^ | 795 ± 363^***^ | 615 ± 268^***^ | 0.2 |
|  | Proportion urinary excretion (%) | 2.7 ± 5.0 | 2.8 ± 5.5 | 2.6 ± 3.7 | 0.9 |
| **Guanidinoacetate** | |  |  |  |  |
|  | Urinary excretion rate (μmol/24-h) | 10.8 ± 7.1^*^ | 10.5 ± 7.2^*^ | 11.5 ± 7.2 | 0.7 |
|  | Dialysate excretion rate (μmol/24-h) | 15.1 ± 8.0^*^ | 16.7 ± 8.2^*^ | 11.1 ± 6.3 | 0.07 |
|  | Proportion urinary excretion (%) | 42 ± 21 | 38 ± 20 | 51 ± 21 | 0.1 |
| **Creatine** | |  |  |  |  |
|  | Urinary excretion rate (μmol/24-h) | 719 [399 – 1070] | 655 [397 – 1015] ^***^ | 867 [464 – 1363] ^***^ | 0.2 |
|  | Dialysate excretion rate (μmol/24-h) | 79 [19 – 196] ^***^ | 58 [5 – 144] ^***^ | 147 [42 – 231] ^***^ | 0.04 |
|  | Proportion urinary excretion (%) | 612 [365 – 902] ^***^ | 545 [365 – 871] ^***^ | 697 [413 – 1052] ^***^ | 0.4 |
| **Creatinine** | |  |  |  |  |
|  | Urinary excretion rate (mmol/24-h) | 3.6 ± 2.0^**^ | 3.5 ± 2.2^*^ | 4.0 ± 1.6 | 0.6 |
|  | Dialysate excretion rate (mmol/24-h) | 6.3 ± 4.1^**^ | 7.0 ± 4.5^*^ | 4.2 ± 1.6 | 0.01 |
|  | Proportion urinary excretion (%) | 38 ± 18 | 34 ± 18 | 48 ± 14 | 0.4 |
| Differences between males and females are tested using an independent sample t-test or Mann-Whitney U test. Differences between extracellular and intracellular removal are tested using paired sample t-test or the paired samples Wilcoxon test, and statistical significance is indicated with asterixis (^*^ P<0.05; ^**^ P<0.01; ^***^ P<0.001). | | | | | |

| **Table S4.** Sensitivity analyses on the associations of predialysis plasma concentrations with characteristics of the protein energy wasting phenotype, in which patients with plasma creatine values above the 95^th^ percentile were excluded. | | | | | |
| --- | --- | --- | --- | --- | --- |
|  | | **Sex-adjusted analyses** | | **Multivariable analyses** | |
|  | | **OR (95% CI)** | **P value** | **OR (95% CI)** | **P value** |
| **Plasma arginine** (per 1-SD decrease) | | |  |  |  |
|  | Low muscle mass | 1.56 [0.77 – 3.76] | 0.4 | 1.53 [0.69 – 4.37] | 0.4 |
|  | Low protein intake | 1.86 [1.03 – 3.79] | 0.06 | 1.83 [0.96 – 3.99] | 0.09 |
|  | Hypoalbuminemia | 2.43 [1.30 – 5.20] | **0.01** | 2.82 [1.37 – 6.98] | **0.01** |
|  | Low BMI | 1.07 [0.59 – 1.89] | 0.8 | 0.92 [0.32 – 2.37] | 0.9 |
|  | Severe fatigue | 1.12 [0.61 – 2.05] | 0.7 | 1.02 [0.48 – 2.15] | 0.9 |
| **Plasma guanidinoacetate** (per 1-SD decrease) | | |  |  |  |
|  | Low muscle mass | 1.34 [0.68 – 3.13] | 0.5 | 1.05 [0.50 – 2.63] | 0.9 |
|  | Low protein intake | 1.59 [0.90 -3.16] | 0.1 | 1.20 [0.63 – 2.52] | 0.6 |
|  | Hypoalbuminemia | 1.48 [0.83 – 2.76] | 0.2 | 1.34 [0.71 – 2.67] | 0.4 |
|  | Low BMI | 1.32 [0.74 – 2.43] | 0.3 | 1.77 [0.77 – 4.39] | 0.2 |
|  | Severe fatigue | 1.18 [0.67 – 2.10] | 0.6 | 0.95 [0.49 – 1.82] | 0.9 |
| **Plasma creatine** (per halving) | | |  |  |  |
|  | Low muscle mass | 1.74 [0.77 – 4.28] | 0.2 | 2.06 [0.77 – 6.47] | 0.2 |
|  | Low protein intake | 2.60 [1.26 – 5.97] | **0.01** | 2.96 [1.26 – 8.26] | **0.02** |
|  | Hypoalbuminemia | 3.02 [1.35 – 7.88] | **0.01** | 6.16 [2.10 – 24.7] | **0.003** |
|  | Low BMI | 0.63 [0.29 – 1.33] | 0.2 | 0.57 [0.18 – 1.58] | 0.3 |
|  | Severe fatigue | 3.11 [1.41 – 7.92] | **0.009** | 3.62 [1.43 – 11.6] | **0.01** |
| **Plasma creatinine** (per 1-SD decrease) | | |  |  |  |
|  | Low muscle mass | 6.64 [2.05 – 32.1] | **0.006** | 4.37 [1.27 – 29.2] | 0.06 |
|  | Low protein intake | 1.45 [0.84 – 2.73] | 0.2 | 1.73 [0.88 – 3.72] | 0.1 |
|  | Hypoalbuminemia | 2.51 [1.33 – 5.49] | **0.009** | 2.47 [1.22 – 5.72] | **0.02** |
|  | Low BMI | 1.71 [0.96 – 3.28] | 0.08 | 2.29 [0.83 – 8.67] | 0.2 |
|  | Severe fatigue | 1.07 [0.60 – 1.89] | 0.8 | 1.57 [0.74 – 3.38] | 0.2 |
| Plasma refers to plasma values predialysis. Analyses of plasma arginine, guanidinoacetate and creatinine are performed on scaled data, with the odds ratio presented per standard deviation decrease of the biomarker. Analyses of plasma creatine are performed on log_2_ transformed data, with the odds ratio presented per halving of the biomarker.  Low muscle mass is defined as a muscle mass <10.76 kg/m2 in males <6.76 kg/m2 in females. Low protein intake is defined as a protein intake <0.8 g/kg/24-h. Low BMI is defined as a BMI <23 kg/m2. Hypoalbuminemia is defined as a serum albumin <38 g/L. Severe fatigue is defined as a subjective fatigue score ≥35. Multivariable analyses are adjusted for sex, age, body surface area, dialysis vintage and hs-CRP. | | | | | |

| **Table S5.** Sensitivity analyses on the associations of predialysis plasma concentrations with characteristics of the protein energy wasting phenotype, in which we adjusted for vascular access type. | | | | | |
| --- | --- | --- | --- | --- | --- |
|  | | **Sex-adjusted analyses** | | **Multivariable (including vascular access type)** | |
|  | | **OR (95% CI)** | **P value** | **OR (95% CI)** | **P value** |
| **Plasma arginine** (per 1-SD decrease) | | |  |  |  |
|  | Low muscle mass | 1.65 [0.84 – 3.89] | 0.2 | 1.48 [0.68 – 3.80] | 0.4 |
|  | Low protein intake | 1.94 [1.08 – 3.94] | **0.04** | 2.11 [1.12 – 4.60] | **0.04** |
|  | Hypoalbuminemia | 2.50 [1.35 – 5.33] | **0.007** | 2.51 [1.21 – 6.10] | **0.02** |
|  | Low BMI | 1.06 [0.59 – 1.86] | 0.8 | 0.84 [0.42 – 1.61] | 0.6 |
|  | Severe fatigue | 1.14 [0.63 – 2.08] | 0.7 | 1.13 [0.60 – 2.14] | 0.7 |
| **Plasma guanidinoacetate** (per 1-SD decrease) | | |  |  |  |
|  | Low muscle mass | 1.08 [0.59 – 2.19] | 0.8 | 0.89 [0.43 – 1.93] | 0.7 |
|  | Low protein intake | 1.55 [0.89 – 2.94] | 0.2 | 1.64 [0.90 – 3.28] | 0.1 |
|  | Hypoalbuminemia | 1.45 [0.82 – 2.65] | 0.2 | 1.48 [0.73 – 3.09] | 0.3 |
|  | Low BMI | 1.17 [0.66 – 2.08] | 0.6 | 1.12 [0.57 – 2.13] | 0.7 |
|  | Severe fatigue | 1.20 [0.69 – 2.15] | 0.5 | 1.15 [0.61 – 2.23] | 0.7 |
| **Plasma creatine** (per halving) | | |  |  |  |
|  | Low muscle mass | 2.00 [1.05 – 4.14] | **0.04** | 2.20 [1.03 – 5.30] | **0.05** |
|  | Low protein intake | 2.13 [1.17 – 4.27] | **0.02** | 2.70 [1.37 – 6.07] | **0.01** |
|  | Hypoalbuminemia | 3.13 [1.46 – 8.02] | **0.008** | 4.36 [1.66 – 15.3] | **0.008** |
|  | Low BMI | 0.74 [0.40 – 1.35] | 0.3 | 0.60 [0.29 – 1.18] | 0.2 |
|  | Severe fatigue | 3.20 [1.52 – 8.05] | **0.006** | 3.63 [1.59 – 10.6] | **0.007** |
| **Plasma creatinine** (per 1-SD decrease) | | |  |  |  |
|  | Low muscle mass | 6.66 [2.14 – 28.6] | **0.003** | 7.13 [1.91 – 41.7] | **0.01** |
|  | Low protein intake | 1.35 [0.80 – 2.43] | 0.3 | 1.31 [0.73 – 2.50] | 0.4 |
|  | Hypoalbuminemia | 2.51 [1.35 – 5.41] | **0.006** | 2.51 [1.18 – 6.09] | **0.02** |
|  | Low BMI | 1.80 [1.02 – 3.45] | **0.05** | 1.71 [0.87 – 3.65] | 0.1 |
|  | Severe fatigue | 1.09 [0.62 – 1.90] | 0.8 | 1.25 [0.66 – 2.40] | 0.5 |
| Plasma refers to plasma values predialysis. Analyses of plasma arginine, guanidinoacetate and creatinine are performed on scaled data, with the odds ratio presented per standard deviation decrease of the biomarker. Analyses of plasma creatine are performed on log_2_ transformed data, with the odds ratio presented per halving of the biomarker.  Low muscle mass is defined as a muscle mass <10.76 kg/m2 in males <6.76 kg/m2 in females. Low protein intake is defined as a protein intake <0.8 g/kg/24-h. Low BMI is defined as a BMI <23 kg/m2. Hypoalbuminemia is defined as a serum albumin <38 g/L. Severe fatigue is defined as a subjective fatigue score ≥35. Multivariable analyses are adjusted for sex, age and vascular access type. | | | | | |

| **Table S6.** Sensitivity analyses on the associations of predialysis plasma concentrations with characteristics of the protein energy wasting phenotype, in which we adjusted for hemodialysis adequacy (Kt/V). | | | | | |
| --- | --- | --- | --- | --- | --- |
|  | | **Sex-adjusted analyses** | | **Multivariable  (including Kt/V)** | |
|  | | **OR (95% CI)** | **P value** | **OR (95% CI)** | **P value** |
| **Plasma arginine** (per 1-SD decrease) | | |  |  |  |
|  | Low muscle mass | 1.65 [0.84 – 3.89] | 0.2 | 2.20 [0.92 – 6.70] | 0.1 |
|  | Low protein intake | 1.94 [1.08 – 3.94] | **0.04** | 2.05 [1.04 – 4.80] | 0.06 |
|  | Hypoalbuminemia | 2.50 [1.35 – 5.33] | **0.007** | 2.23 [1.11 – 5.25] | **0.04** |
|  | Low BMI | 1.06 [0.59 – 1.86] | 0.8 | 1.23 [0.64 – 2.36] | 0.5 |
|  | Severe fatigue | 1.14 [0.63 – 2.08] | 0.7 | 1.05 [0.52 – 2.05] | 0.9 |
| **Plasma guanidinoacetate** (per 1-SD decrease) | | |  |  |  |
|  | Low muscle mass | 1.08 [0.59 – 2.19] | 0.8 | 0.99 [0.47 – 2.68] | 0.9 |
|  | Low protein intake | 1.55 [0.89 – 2.94] | 0.2 | 1.43 [0.71 – 3.68] | 0.4 |
|  | Hypoalbuminemia | 1.45 [0.82 – 2.65] | 0.2 | 1.83 [0.88 – 4.63] | 0.1 |
|  | Low BMI | 1.17 [0.66 – 2.08] | 0.6 | 0.58 [0.11 – 1.42] | 0.3 |
|  | Severe fatigue | 1.20 [0.69 – 2.15] | 0.5 | 1.50 [0.70 – 3.51] | 0.3 |
| **Plasma creatine** (per halving) | | |  |  |  |
|  | Low muscle mass | 2.00 [1.05 – 4.14] | **0.04** | 3.76 [1.54 – 12.0] | **0.01** |
|  | Low protein intake | 2.13 [1.17 – 4.27] | **0.02** | 2.53 [1.25 – 5.90] | **0.02** |
|  | Hypoalbuminemia | 3.13 [1.46 – 8.02] | **0.008** | 5.22 [1.89 – 20.1] | **0.005** |
|  | Low BMI | 0.74 [0.40 – 1.35] | 0.3 | 0.75 [0.37 – 1.47] | 0.4 |
|  | Severe fatigue | 3.20 [1.52 – 8.05] | **0.006** | 3.14 [1.41 – 8.36] | **0.01** |
| **Plasma creatinine** (per 1-SD decrease) | | |  |  |  |
|  | Low muscle mass | 6.66 [2.14 – 28.6] | **0.003** | 5.03 [1.49 – 26.2] | **0.03** |
|  | Low protein intake | 1.35 [0.80 – 2.43] | 0.3 | 1.76 [0.93 – 3.82] | 0.1 |
|  | Hypoalbuminemia | 2.51 [1.35 – 5.41] | **0.006** | 6.03 [2.22 – 23.7] | **0.002** |
|  | Low BMI | 1.80 [1.02 – 3.45] | **0.05** | 2.00 [1.02 – 4.59] | 0.06 |
|  | Severe fatigue | 1.09 [0.62 – 1.90] | 0.8 | 1.17 [0.61 – 2.25] | 0.6 |
| Plasma refers to plasma values predialysis. Analyses of plasma arginine, guanidinoacetate and creatinine are performed on scaled data, with the odds ratio presented per standard deviation decrease of the biomarker. Analyses of plasma creatine are performed on log_2_ transformed data, with the odds ratio presented per halving of the biomarker.  Low muscle mass is defined as a muscle mass <10.76 kg/m2 in males <6.76 kg/m2 in females. Low protein intake is defined as a protein intake <0.8 g/kg/24-h. Low BMI is defined as a BMI <23 kg/m2. Hypoalbuminemia is defined as a serum albumin <38 g/L. Severe fatigue is defined as a subjective fatigue score ≥35. Multivariable analyses are adjusted for sex, age and Kt/V | | | | | |

| **Table S7.** Sensitivity analyses on the associations of predialysis plasma concentrations with characteristics of the protein energy wasting phenotype, in which we adjusted for medication usage | | | | | |
| --- | --- | --- | --- | --- | --- |
|  | | **Sex-adjusted analyses** | | **Multivariable  (including medication)** | |
|  | | **OR (95% CI)** | **P value** | **OR (95% CI)** | **P value** |
| **Plasma arginine** (per 1-SD decrease) | | |  |  |  |
|  | Low muscle mass | 1.65 [0.84 – 3.89] | 0.2 | 1.58 [0.73 – 4.25] | 0.3 |
|  | Low protein intake | 1.94 [1.08 – 3.94] | **0.04** | 1.75 [0.95 – 3.58] | 0.09 |
|  | Hypoalbuminemia | 2.50 [1.35 – 5.33] | **0.007** | 3.34 [1.59 – 8.67] | **0.004** |
|  | Low BMI | 1.06 [0.59 – 1.86] | 0.8 | 1.31 [0.70 – 2.44] | 0.4 |
|  | Severe fatigue | 1.14 [0.63 – 2.08] | 0.7 | 1.05 [0.56 – 1.96] | 0.9 |
| **Plasma guanidinoacetate** (per 1-SD decrease) | | |  |  |  |
|  | Low muscle mass | 1.08 [0.59 – 2.19] | 0.8 | 0.99 [0.51 – 2.08] | 0.9 |
|  | Low protein intake | 1.55 [0.89 – 2.94] | 0.2 | 1.48 [0.83 – 2.87] | 0.2 |
|  | Hypoalbuminemia | 1.45 [0.82 – 2.65] | 0.2 | 1.53 [0.84 – 3.00] | 0.2 |
|  | Low BMI | 1.17 [0.66 – 2.08] | 0.6 | 1.38 [0.76 – 2.56] | 0.3 |
|  | Severe fatigue | 1.20 [0.69 – 2.15] | 0.5 | 1.16 [0.64 – 2.11] | 0.6 |
| **Plasma creatine** (per halving) | | |  |  |  |
|  | Low muscle mass | 2.00 [1.05 – 4.14] | **0.04** | 2.59 [1.20 – 6.50] | **0.02** |
|  | Low protein intake | 2.13 [1.17 – 4.27] | **0.02** | 2.32 [1.14 – 5.47] | **0.03** |
|  | Hypoalbuminemia | 3.13 [1.46 – 8.02] | **0.008** | 8.12 [2.41 – 41.8] | **0.003** |
|  | Low BMI | 0.74 [0.40 – 1.35] | 0.3 | 0.83 [0.42 – 1.70] | 0.60 |
|  | Severe fatigue | 3.20 [1.52 – 8.05] | **0.006** | 4.70 [1.78 – 17.1] | **0.006** |
| **Plasma creatinine** (per 1-SD decrease) | | |  |  |  |
|  | Low muscle mass | 6.66 [2.14 – 28.6] | **0.003** | 5.81 [1.81 – 28.9] | **0.01** |
|  | Low protein intake | 1.35 [0.80 – 2.43] | 0.3 | 1.33 [0.75 – 2.59] | 0.3 |
|  | Hypoalbuminemia | 2.51 [1.35 – 5.41] | **0.006** | 2.77 [1.39 – 6.86] | **0.01** |
|  | Low BMI | 1.80 [1.02 – 3.45] | **0.05** | 2.05 [1.11 – 4.12] | **0.03** |
|  | Severe fatigue | 1.09 [0.62 – 1.90] | 0.8 | 1.09 [0.60 – 1.98] | 0.8 |
| Plasma refers to plasma values predialysis. Analyses of plasma arginine, guanidinoacetate and creatinine are performed on scaled data, with the odds ratio presented per standard deviation decrease of the biomarker. Analyses of plasma creatine are performed on log_2_ transformed data, with the odds ratio presented per halving of the biomarker.  Low muscle mass is defined as a muscle mass <10.76 kg/m2 in males <6.76 kg/m2 in females. Low protein intake is defined as a protein intake <0.8 g/kg/24-h. Low BMI is defined as a BMI <23 kg/m2. Hypoalbuminemia is defined as a serum albumin <38 g/L. Severe fatigue is defined as a subjective fatigue score ≥35. Multivariable analyses are adjusted for sex, age, and the usage of erythropoietin-stimulating agents, vitamin D analogues and corticosteroids. | | | | | |

| **Table S8.** Sensitivity analyses on the associations of predialysis plasma concentrations with characteristics of the protein energy wasting phenotype, in which we adjusted for residual diuresis | | | | | |
| --- | --- | --- | --- | --- | --- |
|  | | **Sex-adjusted analyses** | | **Multivariable  (including residual diuresis)** | |
|  | | **OR (95% CI)** | **P value** | **OR (95% CI)** | **P value** |
| **Plasma arginine** (per 1-SD decrease) | | |  |  |  |
|  | Low muscle mass | 1.65 [0.84 – 3.89] | 0.2 | 1.80 [0.84 – 4.60] | 0.2 |
|  | Low protein intake | 1.94 [1.08 – 3.94] | **0.04** | 2.28 [1.18 – 5.05] | **0.02** |
|  | Hypoalbuminemia | 2.50 [1.35 – 5.33] | **0.007** | 2.88 [1.44 – 6.90] | **0.007** |
|  | Low BMI | 1.06 [0.59 – 1.86] | 0.8 | 1.08 [0.60 – 1.88] | 0.8 |
|  | Severe fatigue | 1.14 [0.63 – 2.08] | 0.7 | 1.18 [0.64 – 2.17] | 0.6 |
| **Plasma guanidinoacetate** (per 1-SD decrease) | | |  |  |  |
|  | Low muscle mass | 1.08 [0.59 – 2.19] | 0.8 | 1.14 [0.58 – 2.45] | 0.7 |
|  | Low protein intake | 1.55 [0.89 – 2.94] | 0.2 | 1.96 [1.01 – 4.23] | 0.06 |
|  | Hypoalbuminemia | 1.45 [0.82 – 2.65] | 0.2 | 1.53 [0.83 – 3.11] | 0.2 |
|  | Low BMI | 1.17 [0.66 – 2.08] | 0.6 | 1.19 [0.67 – 2.14] | 0.5 |
|  | Severe fatigue | 1.20 [0.69 – 2.15] | 0.5 | 1.23 [0.70 – 2.24] | 0.5 |
| **Plasma creatine** (per halving) | | |  |  |  |
|  | Low muscle mass | 2.00 [1.05 – 4.14] | **0.04** | 2.43 [1.16 – 5.81] | **0.03** |
|  | Low protein intake | 2.13 [1.17 – 4.27] | **0.02** | 2.70 [1.32 – 6.47] | **0.01** |
|  | Hypoalbuminemia | 3.13 [1.46 – 8.02] | **0.008** | 4.93 [1.92 – 17.2] | **0.004** |
|  | Low BMI | 0.74 [0.40 – 1.35] | 0.3 | 0.72 [0.38 – 1.31] | 0.3 |
|  | Severe fatigue | 3.20 [1.52 – 8.05] | **0.006** | 3.18 [1.49 – 8.15] | **0.007** |
| **Plasma creatinine** (per 1-SD decrease) | | |  |  |  |
|  | Low muscle mass | 6.66 [2.14 – 28.6] | **0.003** | 12.6 [2.81 – 111] | **0.006** |
|  | Low protein intake | 1.35 [0.80 – 2.43] | 0.3 | 1.60 [0.88 – 3.28] | 0.2 |
|  | Hypoalbuminemia | 2.51 [1.35 – 5.41] | **0.006** | 2.90 [1.46 – 6.89] | **0.006** |
|  | Low BMI | 1.80 [1.02 – 3.45] | **0.05** | 2.09 [1.13 – 4.37] | **0.03** |
|  | Severe fatigue | 1.09 [0.62 – 1.90] | 0.8 | 1.19 [0.66 – 2.17] | 0.5 |
| Plasma refers to plasma values predialysis. Analyses of plasma arginine, guanidinoacetate and creatinine are performed on scaled data, with the odds ratio presented per standard deviation decrease of the biomarker. Analyses of plasma creatine are performed on log_2_ transformed data, with the odds ratio presented per halving of the biomarker.  Low muscle mass is defined as a muscle mass <10.76 kg/m2 in males <6.76 kg/m2 in females. Low protein intake is defined as a protein intake <0.8 g/kg/24-h. Low BMI is defined as a BMI <23 kg/m2. Hypoalbuminemia is defined as a serum albumin <38 g/L. Severe fatigue is defined as a subjective fatigue score ≥35. Multivariable analyses are adjusted for sex, age, and the presence of residual diuresis (yes vs no). | | | | | |

| **Table S9.** Associations of dialysate losses of arginine, guanidinoacetate, creatine and creatinine with characteristics of the protein energy wasting phenotype. | | | | | |
| --- | --- | --- | --- | --- | --- |
|  | | **Sex-adjusted analyses** | | **Multivariable analyses** | |
|  | | **OR (95% CI)** | **P value** | **OR (95% CI)** | **P value** |
| **Dialysate losses of arginine** (per halving) | | |  |  |  |
|  | Low muscle mass | 0.85 [0.49 – 1.51] | 0.52 | 0.76 [0.37 – 1.30] | 0.33 |
|  | Low protein intake | 0.87 [0.46 – 1.42] | 0.60 | 0.75 [0.30 – 1.32] | 0.44 |
|  | Hypoalbuminemia | 1.32 [0.80 – 2.57] | 0.29 | 1.29 [0.72 – 2.28] | 0.32 |
|  | Low BMI | 1.00 [0.55 – 1.67] | 0.99 | 1.31 [0.47 – 2.50] | 0.45 |
|  | Severe fatigue | 0.90 [0.44 – 1.48] | 0.70 | 0.82 [0.43 – 1.56] | 0.77 |
| **Dialysate losses of guanidinoacetate** (per halving) | | | |  |  |
|  | Low muscle mass | 1.32 [0.72 – 2.76] | 0.41 | 0.98 [0.48 – 2.17] | 0.96 |
|  | Low protein intake | 1.41 [0.85 – 2.51] | 0.21 | 0.93 [0.48 – 1.80] | 0.82 |
|  | Hypoalbuminemia | 1.83 [1.08 – 3.30] | **0.03** | 1.37 [0.72 – 2.64] | 0.34 |
|  | Low BMI | 0.91 [0.50 – 1.57] | 0.75 | 1.23 [0.50 – 3.00] | 0.64 |
|  | Severe fatigue | 1.34 [0.74 – 2.49] | 0.34 | 1.18 [0.59 – 2.40] | 0.64 |
| **Dialysate losses of creatine** (per halving) | | |  |  |  |
|  | Low muscle mass | 2.16 [1.28 – 6.31] | **0.02** | 2.41 [1.09 – 6.38] | **0.04** |
|  | Low protein intake | 1.75 [0.97 – 3.37] | 0.07 | 1.70 [0.87 – 3.51] | 0.13 |
|  | Hypoalbuminemia | 4.77 [2.03 – 13.8] | **0.001** | 6.35 [2.17 – 26.6] | **0.003** |
|  | Low BMI | 1.04 [0.56 – 1.97] | 0.91 | 0.81 [0.26 – 2.35] | 0.71 |
|  | Severe fatigue | 2.80 [1.34 – 6.65] | **0.01** | 3.59 [1.52 – 11.0] | **0.01** |
| **Dialysate losses of creatinine** (per 1-SD decrease) | | | |  |  |
|  | Low muscle mass | 12.4 [3.4 – 72.5] | **<0.001** | 11.1 [2.39 – 102] | **0.01** |
|  | Low protein intake | 1.39 [0.80 – 2.55] | 0.26 | 1.77 [0.78 – 4.32] | 0.18 |
|  | Hypoalbuminemia | 3.75 [1.79 – 9.66] | **0.002** | 3.83 [1.48 – 12.3] | **0.01** |
|  | Low BMI | 2.88 [1.45 – 6.71] | **0.006** | 6.28 [1.22 – 74.3] | 0.07 |
|  | Severe fatigue | 1.01 [0.55 – 1.83] | 0.97 | 2.31 [0.95 – 6.20] | 0.07 |
| Low muscle mass is defined as a muscle mass < 0.76 kg/m2 in males <6.76 kg/m2 in females. Low protein intake is defined as a protein intake <0.8 g/kg/24-h. Low BMI is defined as a BMI <23 kg/m2. Hypoalbuminemia is defined as a serum albumin <38 g/L. Severe fatigue is defined as a subjective fatigue score ≥35. Multivariable analyses are adjusted for sex, age, body surface area, dialysis vintage and hs-CRP. | | | | | |

| **Table S10. An overview of the used formulas** |
| --- |
| **Kt/V = – ln (R – 0.008 * t) + (4 – 3.5 * R) * UF/W**  in which R is the ratio between the post- and predialysis concentration of urea, t is duration of the hemodialysis session (h), UF is the ultrafiltration volume (L) and W the body weight after hemodialysis (kg). |
| **Estimated extracellular losses (μmol) = P_X predialysis_ * ECV_predialysis_ – P_X postdialysis_ * ECV_postdialysis_.**  P_X predialysis_ and P_X postdialysis_ refer to predialysis and postdialysis plasma concentrations of arginine, guanidinoacetate, creatine and creatinine, respectively. ECV_predialysis_ and ECV_postdialysis_ were calculated using the formula by Abraham et al. (2011), defined as the square root of pre- or postdialysis body weight, multiplied by height. |
| **Dialysate excretion rate (μmol/24-h) = (V_Dialysate_ * D_X_ * n) / 7. ).**  In this formula V_Dialysate_ refers to the total volume of dialysate (L), D_X_ refers to the measured concentration of arginine, guanidinoacetate, creatine and creatinine in the collected dialysate (μmol/L), and n refers to the number of dialysis sessions per week. |
| **Urinary excretion rate (μmol/24-h) = (Urinary excretion_day 1_ + Urinary excretion_day 2_) / 2.** |
| **Creatinine excretion rate (mmol/24-h) = ((V_Dialysate_ * D_Creatinine_ * n) / 7 + UCrE).**  In this formula V_Dialysate_ refers to the total volume of dialysate (L), D_Creatinine_ refers to measured creatinine concentration in the collected dialysate (mmol/L), n refers number of dialyses per week, and UCrE refers to 24-h urinary creatinine excretion (mmol/24-h), averaged from two 24-h urine collections. |
| **Protein intake (g/kg/24-h) = (6.25 * (0.028 * CUER + 0.031 * BW) + UPE) / BW.**  In this formula CUER refers to combined excretion rate of urea (mmol/24-h), BW refers to body weight postdialysis (kg), and UPE refers to the 24-h urine protein excretion (g/24-h), averaged from two 24-h urine collections. |
| **CUER (in mmol/24-h) = (V_Dialysate_ * D_urea_ * n) / 7 + UUE.**  In this formula, V_Dialysate_ refers total volume of the spent dialysate (L), D_urea_ refers to the measured urea concentration in the collected dialysate (mmol/L), n refers to the number of hemodialysis sessions per week, and UUE refers to the 24-h urinary urea excretion (mmol/24-h), averaged from two 24-h urine collections. |
